# Effect of Indoor Air Pollution on Chronic Obstructive Pulmonary Disease (COPD) Deaths in Southern Asia—A Systematic Review and Meta-Analysis

**DOI:** 10.3390/toxics9040085

**Published:** 2021-04-16

**Authors:** Bellipady Shyam Prasad Shetty, George D’Souza, Mahesh Padukudru Anand

**Affiliations:** 1 Department of Cardiothoracic and Vascular Surgery, Jagadguru Sri Shivarathreeshwara Medical College, Jagadguru Sri Shivarathreeshwara Academy of Higher Education and Research, Mysore 570015, India; nivishyam@gmail.com; 2 Department of Pulmonary Medicine, St John’s National Academy of Health Sciences, Bangalore 560034, India; dsouza.ga1975@gmail.com; 3 Department of Respiratory Medicine, Jagadguru Sri Shivarathreeshwara Medical College, Jagadguru Sri Shivarathreeshwara Academy of Higher Education and Research, Mysore 570015, India

**Keywords:** COPD, chronic obstructive pulmonary disease, South Asia, indoor air pollution, indoor pollution, mortality, death

## Abstract

Background: About half of the population in developing countries are exposed to indoor pollution such as combustion fuels at present. Chronic obstructive pulmonary disease (COPD) is one of the leading causes of mortality globally and the primary cause of COPD in women is indoor air pollution exposure, while tobacco smoking is the leading cause in men. The aim of this systematic review and meta-analysis is to evaluate the correlation between the indoor air pollution and deaths related to COPD and COPD prevalence in South Asia. Methods: A systematic search on studies with sufficient statistical power has been conducted from 1985 until 30 June 2020, in English electronic databases following Preferred Reporting Items for Systematic Reviews and Meta-Analyses guidelines in MEDLINE and PubMed databases with the terms Chronic Obstructive Pulmonary disease COPD or Chronic Bronchitis or Emphysema or COPD Deaths or Chronic Obstructive Lung Disease or Airflow Obstruction or Chronic Airflow Obstruction or Airflow Obstruction, Chronic or Bronchitis, Chronic and Mortality or Death or Deceased was conducted. Studies were eligible if they were Prospective controlled or non-controlled trials conducted in Southern Asia/ Asia and Retrospective studies conducted in Southern Asia/ Asia. Results: The results have concluded that long term exposure to indoor pollution had a significant effect on COPD deaths as well as its symptoms. Odd’s ratio was in a range of 1.05 (Randomized controlled trials) to 7.87 (Cross sectional studies) for all the studies mentioned. Meta-analysis observed a significantly higher Odds Ratio of 2.13 for COPD mortality and 2.08 for COPD prevalence on exposure to indoor air pollution. Conclusion: Exposure to solid fuel smoke is consistently and significantly correlated with COPD mortality and COPD prevalence in South Asian countries, in spite of heterogeneity observed in the studies included. For performing domestic tasks, initiatives are to be taken to reduce dependency on solid fuel by using cleaner alternatives or comparatively cleaner technology.

## 1. Introduction

Chronic obstructive pulmonary disease (COPD) is one of the major causes of death in the developing countries and a leading cause for mortality worldwide. It has always been a major concern for public health and growing health care problem expected to potentially increase affecting all age groups [1]. To meet their basic household energy demands, around half the global population, are exposed to indoor air pollution in the form of solid fuel such as biomass. The large proportion of this exposed population tend to live in less economically developed countries (LEDCs) in particular, south Asian countries [2,3]. These countries generally include India, Pakistan, Bangladesh, Afghanistan, Nepal, Sri Lanka, Bhutan, and Maldives. As per the recent report by International Energy Agency, 2.8 billion people worldwide still lack access to clean cooking fuels. The majority of this population (1.9 billion) is from developing countries of Asia [4]. The Global Initiative for Chronic Obstructive Lung Disease (GOLD) has defined COPD as a common, treatable, and preventable disease state which is characterized by airflow limitation ascribable to airway and/or alveolar abnormalities and persistent respiratory symptoms which are usually caused by significant exposure to noxious particles or gases. Persistent airflow obstruction diagnosed through post-bronchodilator spirometry evaluation (FEV_1_/FVC < 0.70) is determinant of COPD. The severity of airflow obstruction is determined by the forced expiratory volume (FEV1), ≥ 80% is determined to be mild obstruction, whereas < 30% is determined as very severe obstruction [5].

Chronic exposure to wood smoke and other forms of biomass in developing countries is a contributing factor for the rise in prevalence of chronic bronchitis, respiratory failure, and cardiopulmonary diseases [2]. In India and other developing south Asian nations, the prime source of indoor energy are firewood and dung [3]. Particulate matter, comprised of microscopic solids or liquids which are easily inhaled and cause serious health issues, are one of the principal factors for outdoor and indoor air pollution. Studies have evaluated the effects of larger particles such as PM10, but it is the smaller particles which settle deep into the lungs (PM2.5, PM1.0) that are the cause of respiratory illnesses and the ultra-fine particles may even enter the systemic circulation and pose a greater risk to health at distant organs [6].

Indoor air pollution generally produces several pollutants including NO_2_, SO_2_, Nano particles, and Ozone. The composition and particulate matter of biomass smoke is similar to cigarette smoke. It contains harmful elements which damage health such as particulate matter of multiple sizes in high levels, nitrogen oxides, oxides of carbon, formaldehyde, bezene derivatives, polycyclic organic hydrocarbons, including benzopyrene and transitional metals such as Aluminum, Copper, Iron, Nickel, and Zinc [7]. These pollutants are responsible for crossing the alveolar-capillary barrier, penetrating significantly into lungs and cause temporary or permanent pulmonary damage [8]. Utilization of solid biomass fuel for rural domestic energy requirements is a cause of diverse and severe respiratory illnesses, as stated by a meta-analysis of 25 studies [9].

In low- and middle-income countries, due to the demand in daily needs and chronic exposure to indoor smoke, about 35% of patients have developed COPD. In the Global Burden of Disease study done in Indian population on chronic respiratory diseases, the results showed that India has a disproportionately high burden of chronic respiratory diseases [10]. COPD, asthma, and lung cancer are the three main respiratory diseases which are caused due to pollutants. Since women are the main cooks in the family and due to increase in the indoor pollution hazards, they are affected more than men. Over past two decades, studies have shown that COPD mortality rates have increased more in women than in the men [11,12].

There is a paucity of information on the impact of indoor air pollution on COPD mortality and COPD prevalence in South Asian countries. Recent systematic reviews have presented pooled estimates of COPD mortality and prevalence for global and Asia as a whole, but not researched South Asian countries which are developing countries. South Asian countries have the additional burden of poor nutrition, population is endemic for tuberculosis, and exposure to dust is a common occupational hazard [13]. In this background, there is a need to understand whether the effect sizes of indoor air pollution on COPD mortality and COPD prevalence are different in the South Asian countries as compared to global estimates or Asian estimates. Thus, this systematic review and meta-analysis was conducted to highlight the relationship between indoor air pollution effects on COPD prevalence and COPD deaths in South Asian countries.

## 2. Methods

### 2.1. Search Strategy

Systematic search was performed to identify the studies on the associations between indoor pollution and COPD deaths (Table 1). The comprehensive online electronic databases included PubMed, Cochrane database, and Google scholar (Advanced Google search), and we conducted a literature search for terms such as COPD, chronic obstructive lung disease, indoor air pollution, nano-particles, ozone, chronic bronchitis, demise, death, air flow obstruction, south Asia, southern part of Asia, and using “or” and “and” as combining terms for death and indoor air pollution.

### 2.2. Selection of the Studies

The search was restricted to English language. No limitation was set for participant’s age. To better fulfill our objectives, studies who recruited patients who were having COPD and had or were having exposure to indoor pollution and death due to COPD were included in the search. Studies belonging to south Asia countries were particularly searched. Studies which are Prospective controlled or non-controlled trials conducted in Southern Asia/Asia. Retrospective studies conducted in Southern Asia/Asia were included. This systematic review and meta-analysis include studies from only countries of south Asian regions which are highly populated and developing countries.

This review follows guidelines of the Preferred Reporting Items for Systematic Reviews and Meta-Analysis Statement (PRISMA). PRISMA checklist has been attached as a Supplementary Material. The titles and abstracts of all articles were screened by authors BSP and MPA for the first selection process using the eligibility criteria.

Studies excluded were which recruited Patients without Chronic Obstructive Pulmonary disease (COPD), Patients with only data of Outdoor air pollution, Patients who live outside South Asia/Asia. In studies where primary data was not collected, duplicate publications were also not considered for the search. All references of the identified papers were screened for any additional article which has been missed and not been identified in the original search. Two reviewers checked all the titles and abstract and evaluated the full text of potential studies independently.

Particulate matter concentrations i.e., PM 2.5 or PM 10, were considered for exposure measurement in this systematic review.

### 2.3. Quality Assessment

To eliminate any potential bias from combining studies of differing methodologies which might lead to ambiguous conclusions, all included studies were subjected to quality assessment. All the studies were assessed based on clear aims, randomization techniques, eligibility for intervention, blinding of interventionists and participants, description of intervention provided to allow replication, effect size, details of long term follow up and sustained change, analysis of confounding variables, the definition of all outcomes measured with reliable measurement tools. The results provided for each and appropriate statistical analysis were also assessed. Two independent reviewers graded the extracted data quality of each data point (Table 2) as ‘Good’, ‘Poor’, and ‘Not-Assessable’. Disagreements were resolved, if any, through discussion and consensus decision. The grading has been presented in Table 3 and Table 4.

### 2.4. Data Management

Data was extracted based on screening titles, abstracts, and full texts according to the inclusion criteria.

### 2.5. Statistical Analysis

After the initial screening process, full text articles were included for the final and complete review. The data of the studies which were finalized and were ready to be included in the study were entered into an Excel sheet. The data from the respective finalized studies included Odds Ratio values and their corresponding Confidence interval values (95% CIs).

All the statistical analysis and forest plots were generated using the statistical software STATA version 16.0. The values of Odds Ratio’s and Confidence intervals collected were subjected to statistical conversion by applying Log to Odds Ratio’s and 95% CIs into their respective Risk Ratio values and their CIs. These were considered as effect size values. A standard error (SE) has been estimated based on the calculated log values of 95% Confidence Intervals. A funnel plot was generated by obtaining standard error values and the effect size values, whereas the Forest plots were populated based on the collected values of ORs and the estimated 95% CIs based on overall effect size from their respective finalized studies.

## 3. Results

### Characteristics of the Articles

**Figure 1:**
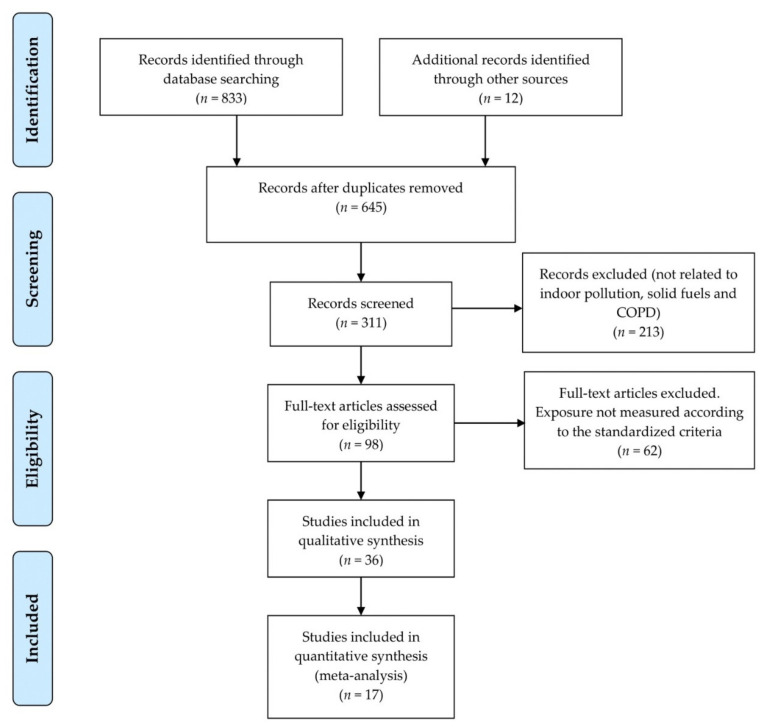
The above flow chart describes the methodology and flow for searching strategy of studies. Preferred Reporting Items for Systematic Reviews and Meta-Analyses (PRISMA) study selection flow chart.

Our search identified 833 citations up to 30 June 2020. After review of title of the article and removal of duplicates, 164 abstracts were reviewed, and 49 articles were retained for full-text review. Some articles did not meet the inclusion criteria and we excluded 46 full text papers that had insufficient data for extraction. Final evaluation yielded 17 eligible papers for inclusion in the study. The detailed selection process of studies is shown in Figure 1.

**Figure 2:**
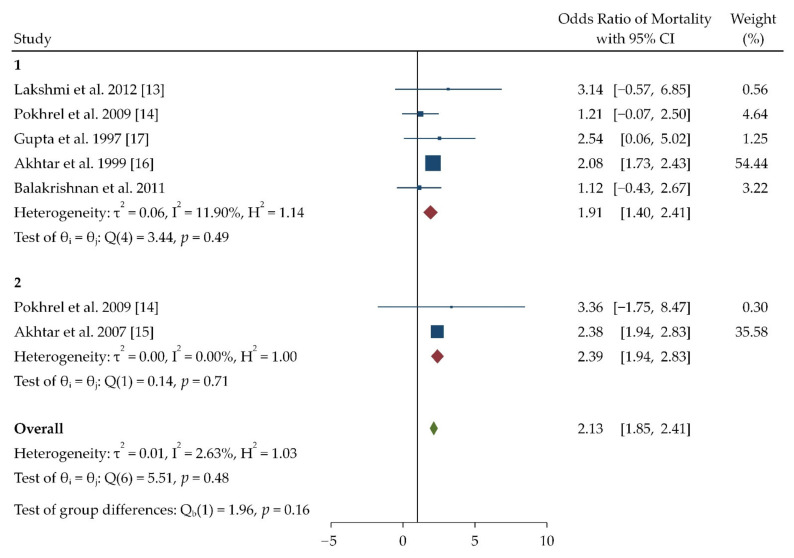
Forest plot of odd’s ratio for chronic obstructive pulmonary disease (COPD) mortality. The plot has been generated using Random effects Restricted maximum likelihood (REML) model. The forest plot has been given in terms f pollutant which have been assessed in respective studies. The number represents the pollutant which the studies have assessed, which is as follows: 1 = Biomass; 2 = Kerosene.

Table 3 summarizes a few important articles which had an acceptable Odds Ratio, and the studies were from India, Nepal, and Pakistan. Database search did not yield any published research from other South Asian countries which matches our systematic review selection criteria. The meta-analysis included 6 studies (Table 3) which focused association between outcomes of COPD (particularly deaths) and indoor pollution. Most of the studies included women as their subjects and a few of the studies included mixed gender recruitment. The effect of indoor pollution on COPD mortality is derived through a forest plot which can be observed in Figure 2.

The forest plot generated for COPD mortality (Figure 2) presents the overall odds ratio and heterogeneity in the studies included for meta-analysis. Two studies, i.e., Akthar et al. in 2007 and in 1999, were found to have higher weightage than other studies (35.58 and 54.55), respectively. The overall heterogeneity observed was 0.01 and 2.63%. The indoor pollutant biomass had the highest weightage according to the study done by Akthar et al. in 1999 [15,16].

**Figure 3:**
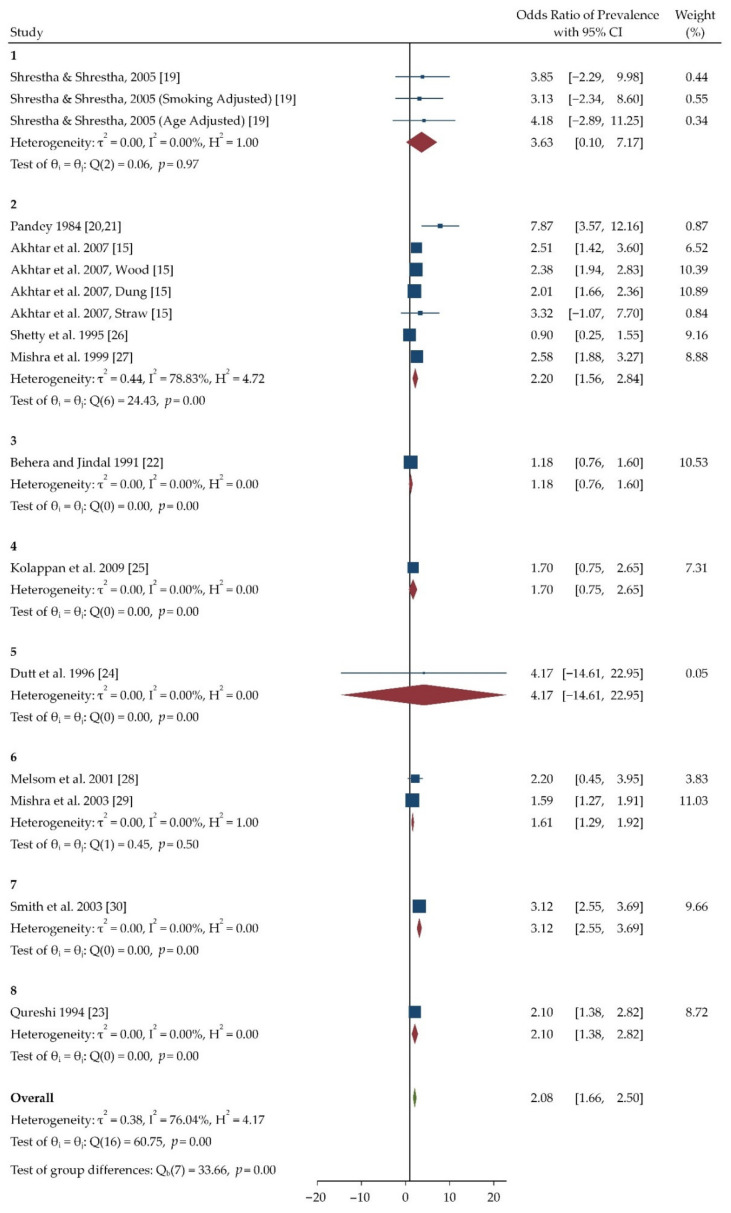
Forest plot of odd’s ratio for COPD prevalence. The plot has been generated using Random-effects Restricted maximum likelihood (REML) model. The forest plot has been given in terms of pollutant which have been assessed in respective studies. The number represents the pollutant which the studies have assessed, which is as follows: 1 = PM10, CO; 2 = Biomass; 3 = Biomass, LPG, Kerosene and Mixed; 4 = Kerosene; 5 = Biomass, LPG and Kerosene; 6 = Biomass and Clean Fuel; 7 = Kerosene and Biomass; 8 = Biomass and Mixed Fuel.

**Figure 4:**
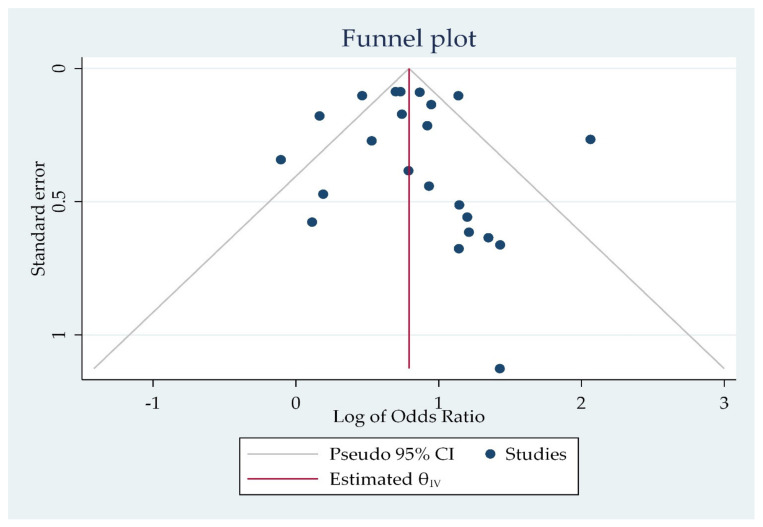
Funnel plot for odd’s ratio values and their ranges.

Table 4 summarizes selected studies which had an acceptable Odds Ratio, and the majority of studies were from Nepal, India, and Pakistan. Twelve studies focused on association between COPD prevalence and short-term exposure of indoor pollutants. The table shows COPD prevalence among the people who use resources like kerosene, biomass, etc. for cooking and household purposes. Most of the studies are cross sectional and case control studies with an odds ratio being in a range of 0.90 to 7.87. Population groups selected in the studies are mostly > 50 years of age. A forest plot was generated based on the collected Odds Ratio data to measure the effect of indoor pollution on COPD prevalence which can be observed in Figure 3. A funnel plot was also generated to check the publication bias which can be observed in Figure 4.

The generated forest plot (Figure 3) shows the odds ratio for individual studies on COPD prevalence as well as overall mean odds ratio and heterogeneity in the studies included for meta-analysis. The highest value for COPD prevalence was seen in the study done by Pandey et al. in 1984 (7.87) in which the exposure was not measured directly. The mean odds ratio observed was 2.08 (1.66, 2.50). The Particulate matter 10 had higher odds ratio according to a study done by Shrestha and Shrestha et al. (4.18). Most of the studies confirmed that women were more prone to COPD than men, with a reported average estimated odds ratio of 1.32 [27,28,30].

## 4. Discussion

In this study we reported the association between indoor pollution and COPD in south Asia, including studies published until mid-2020. The present systematic review and meta-analysis reexamines existing research findings about the association between indoor pollution and COPD deaths in South Asian countries. A total of 17 individual studies were analyzed based on the specific inclusion and exclusion criteria from MEDLINE and PUBMED from 1985 to 30 June 2020. Based on 11 Prospective controlled or non-controlled trials conducted in Southern Asia/Asia and 6 Retrospective studies, this systematic review provides a confirmation that exposure to indoor pollution is related to COPD deaths and prevalence. Most of the studies confirmed that women were more prone to COPD than men with a reported odds ratio of 1.32 [27,28,30]. The studies conducted in various regions of south Asia confirmed that low economic background in some developing countries was one of the main reasons for COPD [31,32]. A study done by Qureshi et al. concluded that indoor pollution like biomass and solid fuels had significant impact on COPD deaths with an odds ratio of 2.0. Risk was relatively higher in rural areas than urban areas (OR = 2.0) [23].

Inclusion of subjects who have been smokers for years or were present smokers in few studies was one of the major limitations of this meta-analysis. Stratification in the studies were done according to smokers and non-smokers. Due to this limitation of including smokers there might have been a lowered significance of the studies, which may have an impact on the results of effect of indoor pollution on COPD mortality and prevalence. While considering COPD and Chronic bronchitis separately, our study found that people who were exposed to indoor pollution had significant changes in lung morphology and histology. Smoking causes an additional impact, and significantly increases the severity and prevalence of COPD in women. Previous studies have calculated a relative risk of about 2.6 in non-smokers than smokers and who are women [33,34,35,36,37].

True risk may vary in the present study because people in developing countries consider wheezing, dyspnea, and phlegm as normal, and can probably result in under-reporting of symptoms [38]. All the studies in this review did not use spirometry as a standard for diagnosing COPD. Chronic respiratory symptoms in the general population were also considered as COPD.

In contrast to this study, a systematic review done by Mc Kay Ailsa in 2012 and Jan et al. did not identify any studies from which they could draw a rigorous estimate of the prevalence of COPD by standard definition [12,39]. Abdo in 2016 stated in their systematic review that adverse health outcomes were strongly associated with various pollutants in the Eastern Mediterranean Region [40]. Kurmi measured respirable dust and PM 2.5 levels in rural and urban areas of Nepal and observed that the PM 2.5 levels showed two peaks in the morning and evening that was associated with cooking with biomass fuels [41]. In accordance with this study, studies done by Kodgule, Pandey and Kroeger also found out that indoor pollution and COPD were strongly associated [42,43,44].

India being a developing country, organic resources such as coal, wood, and cow dung are utilized as primary fuel source indoor for purpose such as cooking. The highest amounts of exposures are seen in women and children. Out of 200 confirmed cases of COPD, 56.5 % of cases were nonsmokers and 43.5% were smokers as per a study conducted by Mahmood et al. [45]. Pandey et al. [43] in his study observed an OR of 2.44 in biomass fuel exposed women in developing COPD, which was lower than the risk observed in this meta-analysis (OR was 3.16).

Studies conducted on similar population (women > 30 years of age) by Smith et al. [30] and Balmes [46] has shown a relative risk of 3.2 (95% CI 2.3–4.8) and 1.78 (95% CI 1.45–2.18), respectively. A meta-analysis of 25 studies on indoor pollution by Po et al. has showed OR of 2.40 (95% CI 1.47–3.93) in the female population [9]. Variation in multiple studies observed was due to variation in fuel quality and the resultant smoke emitted. Moreira et al. studied COPD in Brazilian female population who are exposed to firewood smoke, and it was observed that COPD group has higher years of firewood smoke exposure (P = 0.043) than controls [47]. From the results of the above studies, it can be inferred and concluded that COPD affects women more than men when no additional risk factors are associated with indoor pollution.

This paper gathers 17 papers with different results. The publication research period ranged from 1985 to 2020, representing almost 35 years. In comparison with other previous meta-analysis and systematic reviews, this review had 3 additional papers and results, including studies of Akhtar, Kolappan, and Balakrishnan et al. [15,18,25]. These studies provided a broad risk level analysis about the relation between indoor pollution and COPD. Pathak et al. [48] performed a systematic review in 2020 which is the latest one, drawing association between indoor pollution from biomass cooking and COPD. The review included global regions of Africa, Asia, Europe, North and South America, in which Asian region showed an odds ratio of 2.88 and the review concluded biomass to be associated with COPD. The review considered the whole of Asia rather than segregating the Asian countries. Evaluation of the results was also broad and diversified in this review.

Additional criteria with well-designed morphological and histological assessment of lung and specific particulate matter (PM2.5/10) effecting COPD should be included to confirm the findings from the present systematic review and meta-analysis.

### 4.1. Interpretation of Findings in Relation to Previously Published Work

To the best of our knowledge, a systematic review on COPD mortality and prevalence in Southern Asia has not been performed to date. Previous studies conducted by Jindal et al. observed a mean COPD prevalence of 5% and 2.7% in males and females, respectively [49]. Our review results inferred that COPD rates in South Asia are similar or slightly more than developed regions such as Europe, North America, and potentially lower than some neighboring countries in other parts of Asia. Contrary to this, the analysis of data from the Burden of Obstructive Lung Disease (BOLD) study revealed that COPD prevalence in developed nations is slightly higher than in India [50].

Table 5 summarizes the pooled estimates of the systematic reviews conducted in the past two decades. The overall pooled estimate of present systematic review for COPD mortality is 2.13 (1.85–2.41). This is in accordance with the overall pooled estimates of systematic reviews conducted by Po et al. and Kurmi et al. [9,51]. The observed similarity might be related to compact sample size in published research selected in both reviews, with exclusion of coal as a source of indoor fuel and considered a general definition of COPD. Heterogeneity was also observed in terms of biomass fuels studied and definition of health outcomes in both the reviews. Combustion efficiency varies depending on the fuel source, and wood, charcoal, and animal dung have much lower combustion efficiency than that of non-biomass fuels such as kerosene and LPG. Except for the systematic review by Pathak et al. [48], all other reviews evaluated only COPD mortalities in global population as a whole rather than specific countries.

Another difference noticed in other reviews in contrast to our review is that the confidence intervals of odds ratio of overall pooled data is higher in the other systematic reviews compared to our review. This may be due to narrow selection criteria implemented for our review.

### 4.2. Strengths

This study researched and estimated the chronic effect of particulate matter due to indoor air pollution and its effect on increase of COPD mortality. It has provided a quantitative evidence for Indian government and other LMIC countries in South Asia by explaining the health burden related to indoor pollution and its effect on COPD. In countries which have a developing economy and are being rapidly industrialized, COPD control can be achieved by setting up an air quality plan and implementing it.

### 4.3. Practical Implications

The present study’s findings provided a strong association between short-term indoor air pollution exposure and the risk of COPD exacerbations. These findings can help improve and implement regulations on the air quality that will provide measurable benefits to public health. Our results also provide a way for future research studies on the relationship between indoor air pollutants and the risk estimation.

### 4.4. Limitations of the Study

It was acknowledged that few limitations could be considered. First, the selection of methodologies was a little controversial. Values of pollutants causing COPD should have been standardized. Finally, the air pollutants were considered in combination, and each of the pollutants was not assessed separately, which could have given this review more strength. A single air pollutant method would be preferable for more strength. Dose response function determination was not possible in our review as the studies selected for our review did not provide relevant data.

## 5. Conclusions

This study confirms that indoor pollution is significantly associated COPD deaths, and this systematic review can help assess the risk associated with indoor pollution. Many low-income, middle-income countries face an important issue with the effect of indoor pollution on COPD deaths. There is a need to use clean fuels like LPG, but many households may use clean alongside traditional ones, mainly in urban areas. The present study also concluded that women were more exposed to indoor pollution and were more affected than men. This study also considered the mean odds ratio and confidence intervals to evaluate the overall effect of indoor pollution on COPD. This review presented the pooled estimates for COPD prevalence and COPD mortality in South Asia. The countries in this region have a high burden of poverty, malnutrition, are endemic for infections such as tuberculosis, and a considerable number of the population is exposed to dust as part of their occupation. Our review is a call to the governments to take action and enact a clean air policy. Further studies and additional data are needed to explore further indoor pollution and direct measurements of particulate matter and other pollutants in causing both COPD disease and deaths.

## Figures and Tables

**Table 1:** Participants, interventions, comparisons, outcomes, and study design (PICOS) identifiers from research questions (‘key terms’) used to generate database searches.

	Participants	Interventions	Comparisons	Outcomes		Study Design
Key terms	All population residents in southern Asia	N/A	N/A	Prevalence, COPD deaths, indoor pollution, southern ASIA	(Chronic Obstructive Pulmonary disease (COPD)) or (COPD) or (Chronic Obstructive Pulmonary disease) or (Chronic Bronchitis (CB)) or (Chronic Bronchitis) or (Emphysema) or (COPD Deaths) or (Chronic Obstructive Airway Disease (COAD)) or (Chronic Obstructive Airway Disease) or (COAD) or (Chronic Obstructive Lung Disease) or (Airflow Obstruction) or (Chronic Airflow Obstruction) or (Airflow Obstruction, Chronic) or (Bronchitis, Chronic) and (Mortality) or (Fatality) or (Demise) or (Death) or (Deceased) and (Indoor Air Pollution) or (PM2.5) or (PM10) or (NO_2_) or (SO_2_) or (Nano particles) or (Ozone) and (South Asia) or (Southern Asia) or (Southern Part of Asia) or (Southern Countries of Asia) or (South Asian Countries)	All

**Table 2:** Data extraction and quality assessment checklists. The numbers beside the quality assessment criteria are used to indicate the 8 points.

General Data Extraction	Quality Assessment Check List
Study dates or Publication dates Study design Type of report Number of participants (enrolled, excluded and lost to follow up) Participants characteristics (age, sex, exposure to indoor pollution) Study setting (place/urban/rural) Definition of diagnosis used Measurement/assessment tool Out Comes (Fuel exposure/Odd’s ratio) Outcomes (Prevalence of COPD )Outcomes (COPD mortality)	Type of report Clear aims and objectives Clear and appropriate methods, including sampling and recruitment Appropriate analysis Out comes not reported/additional outcomes reported Risk of bias in selection Risk of bias in measurement and outcomes Limitations discussed

**Table 3:** Odds ratios of studies showing COPD mortality.

First Author, Year, Reference	Study Design	City/Country	No of Subjects Recruited	Exposure Measured	Study Population	Risk Level	Quality
Lakshmi et al. 2012 [13]	Case control study	India	126 cases and 252 controls	Biomass	Age matched; community based	3.14 (1.15–8.56) for COPD Mortality	G = 6 P = 1 N = 2
Pokhrel et al. 2005 [14]	Case control study	Nepal	125 cases and 250 controls	Kerosene, Biomass	Adults	1.21 (0.48–3.05)-biomass for COPD Mortality 3.36 (1.01–11.22)-kerosene for COPD Mortality	G = 10 P = 1 N = 4
Akhtar et al. 2007 [15]	Cross sectional	Pakistan	304 subjects	Kerosene	> 60 years	2.38 (2.12 to 3.01) for COPD Mortality	G = 8 P = 4 N = 1
Akhtar et al. 1999 [16]	Cross sectional	India	212 subjects	Biomass (Dung cake)	> 55 years	2.08 (1.72 to 2.42) for COPD Mortality	G = 7 P = 4 N = 1
Gupta et al. 1997 [17]	Cross sectional studies	India	707	Biomass	Adults	2.54 (1.07–6.04) for COPD Mortality	G = 7 P = 2 N = 3
Balakrishnan et al. 2011 [18]	Case control	India	110 subjects	Biomass	> 20 years	1.12(0.36 to 3.45) for COPD Mortality	G = 7 P = 2 N = 2

N = not assessable; G = good; P = poor

**Table 4:** Odds ratios of studies showing COPD prevalence.

First Author, Year, Reference	Study Design	City/Country	No of Subjects Recruited	Exposure Measured	Study Population	Risk Level	Quality
Shrestha and Shrestha 2005 [19]	Cross-sectional survey	Kathmandu valley, Nepal	168 subjects 94% women 4 municipalities 98 households	PM10, µg/m^3^ CO, µg/m^3^	Age matched; Adults	COPD prevalence, %: Solid fuels 16.8, Clean fuels 7.0 OR for COPD/asthma: Unadjusted 3.85 (1.11–13.38); Smoking adjusted 3.13 (0.83–11.76); Age adjusted 4.18 (1.14–15.27)	G = 8 P = 1 N = 0
Pandey 1984 [20,21]	Cross-sectional study	Nepal	Non-smoking adults > 20 years Sample = 748	Biomass	Adults	13.76% (women) 7.87 (4.67 to 13.26) 3.0% (men) OR = 7.87 (4.67 to 13.26)	G = 7 P = 2 N = 2
Behera and Jindal 1991 [22]	Cross sectional	India	N = 3701 Women	Biomass, LPG, kerosene, mixed	> 50 Years	2.9% (biomass) 1.3 (kerosene), 2.5 (LPG) and 1.2 (mixed) OR = 1.18 (0.83 to 1.67) (biomass user)	G = 5 P = 1 N = 2
Qureshi 1994 [23]	Case control study	India	(MF)560 (MF)286 (BM)Adults >15 years	Biomass (BM), mixed fuel	> 50 years	10.14% (BM), OR = 2.10 (1.50 to 2.94) (biomass user) 5.11% (MF)	G = 7 P = 2 N = 1
Akhtar et al. 2007 [15]	Case control study	Pakistan	1426 cases and 1131 controls Female nonsmokers	Biomass	Adults; age matched	7.01% (cases) and 2.92% (control) 2.51 (1.65 to 3.83) (BM) Wood 2.38 (2.12 to 3.01); Dung 2.01 (1.72 to 2.42); Straw 3.32 (1.11 to 9.88)	G = 6 P = 1 N = 4
Dutt et al. 1996 [24]	Case control study	India	97 (biomass) 100 (kerosene) 98 (LPG) Women	Biomass, LPG and kerosene	> 60 years	4.1% (biomass) 2.0% (kerosene) 1.0% (LPG) OR = 4.17 (0.46 to 38.02) (biomass vs LPG)	G = 7 P = 2 N = 2
Kolappan et al. 2009 [25]	Case control study	India	255 cases and 1275 controls	Kerosene	> 60 years	1.7 (1.0–2.9)	G = 6 P = 1 N = 3
Shetty et al. 1995 [26]	Case control study	India	19 cases and 189 controls women	Biomass	> 60 years	0.90 (0.46–1.76)	G = 8 P = 2 N = 1
Mishra et al. 1999 [27]	Cross sectional study	India	260162 persons screened Women	Biomass	All aged > 20 years in the sampling location	2.58 (1.98–3.37)	G = 5 P = 1 N = 3
Melsom et al. 2001 [28]	Case control	Nepal	121 cases and 126 control women	Biomass and clean fuel	Children aged 11–17 years. ISAAC criteria	2.2 (1.0–4.5)	G = 6 P = 2 N = 2
Mishra et al. 2003 [29]	Cross sectional	India	38959 subjects women	Biomass and clean fuel	Based on interviewing	1.59 (1.30–1.94)	G = 5 P = 2 N = 4
Smith et al. 2003 [30]	Cross sectional	Developing countries	321 subjects women	Kerosene and biomass	> 30 years	3.12 (2.31–3.45)	G = 7 P = 4 N = 2

N = not assessable; G = good; P = poor

**Table 5:** Pooled Estimates of Systematic Reviews showing COPD Prevalence and Mortality in the past two decades.

Systematic Review Study Author Year of Publication	Pooled Estimate Global	Pooled Estimate Asia	Pooled Estimate South Asia	Pooled Estimate Global	Pooled Estimate Asia	Pooled Estimate South Asia
	COPD Prevalence			COPD mortality		
Pathak et al. 2020 [48]	2.65 (2.13–3.31)	2.88 (2.03–4.08)	Not assessed	Not assessed	Not assessed	Not assessed
Sana et al. 2018 [37]	Not assessed	Not assessed	Not assessed	1.20 (0.99 to 1.40)	Not assessed	Not assessed
Kurmi et al. 2010 [51]	Not assessed	Not assessed	Not assessed	2.80, (1.85 to 4.0)	Not assessed	Not assessed
Po et al. 2011 [9]	Not assessed	Not assessed	Not assessed	2.40, (1.47 to 3.93)	Not assessed	Not assessed

## Data Availability

Data sharing not applicable.

## Supplementary Materials

The following are available online at https://www.mdpi.com/article/10.3390/toxics9040085/s1, Supplementary Material: Preferred Reporting Items for Systematic Reviews and Meta-Analyses (PRISMA) Checklist.
